# Supplementary material for: Protection and Restoration of Damaged Hair via a Polyphenol Complex by Promoting Mechanical Strength, Antistatic, and Ultraviolet Protection Properties
Source: Biomimetics (Basel). 2023 Jul 9;8(3):296. doi: 10.3390/biomimetics8030296 (PMC10807499; doi:10.3390/biomimetics8030296)
Supplement: Supplementary file 1 [file biomimetics-08-00296-s001.zip › biomimetics-2457673-supplementary.pdf]

1. Experimental Section

1.1. In vitro investigation of ROS scavenging capabilities

For optical imaging, HeLa cells with a concentration of 10^5 cells/well were cultured on a 24-well plate and placed in a humidified incubator (5 % CO_2 atmosphere) for 12 h at 37°C. Then, the solution media was changed with media containing TA/GA/CA at a concentration of 1.6 mg/mL and incubated at designated times (0, 3, 6, and 12 h). The cells were detached using trypsin-EDTA, centrifuged, and washed with PBS pH 7.4 before being stained with 2′,7′-dichlorodihydrofluorescein diacetate (H2DCFDA) and imaged with a confocal microscope (magnification, 40×).

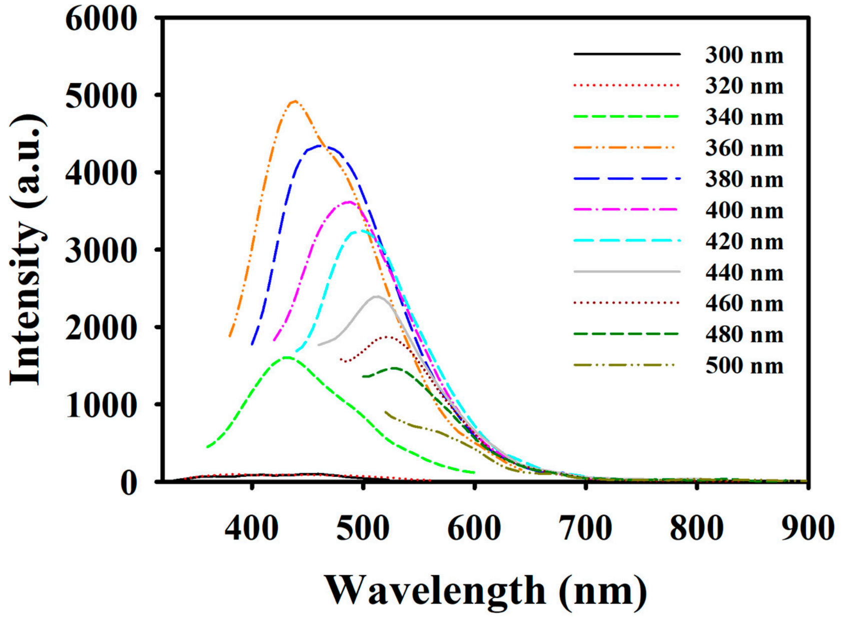

Figure S1. The photoluminescence (PL) spectra of PPC1.

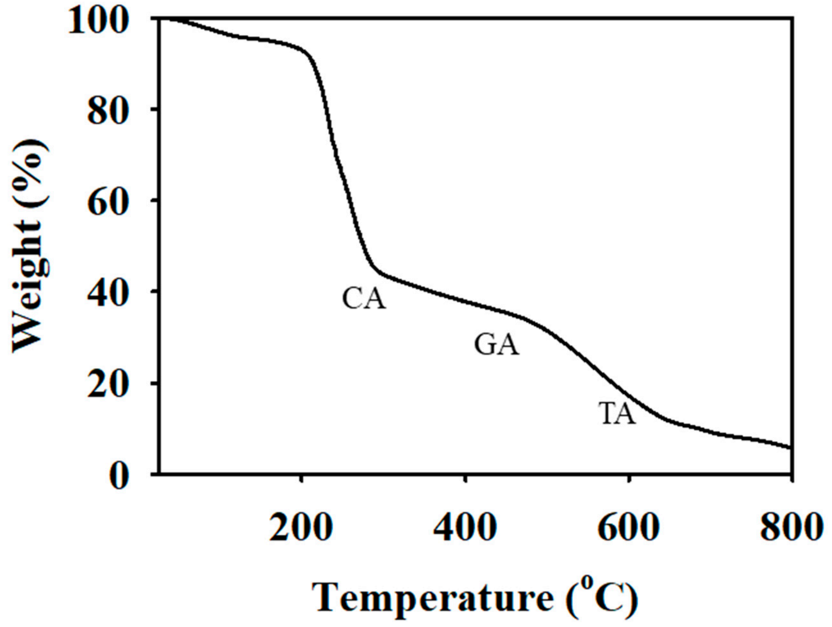

Figure S2. The thermogravimetric analysis (TGA) profile of PPC1.

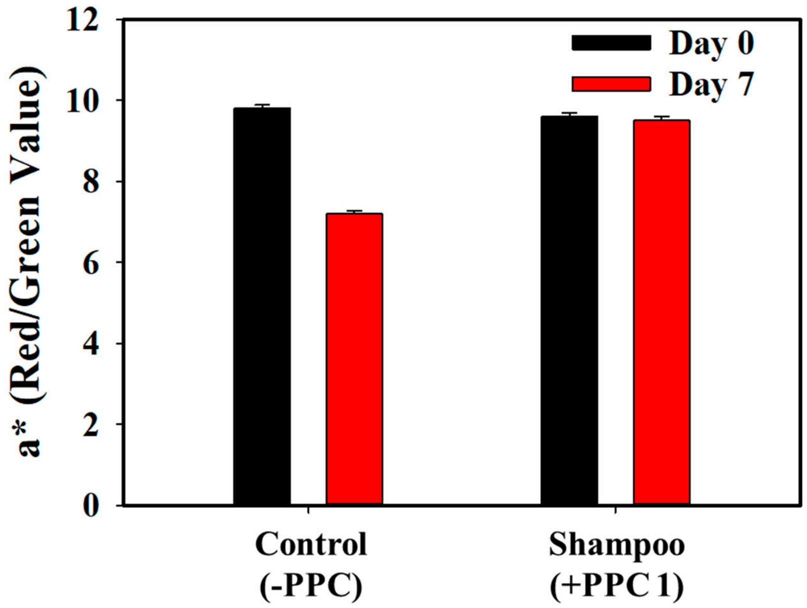

Figure S3. The change of color (a*, red and green) on dye-coated hair model treated with control (shampoo without PPC) and shampoo + PPC1 after 7 days exposed by UV light.

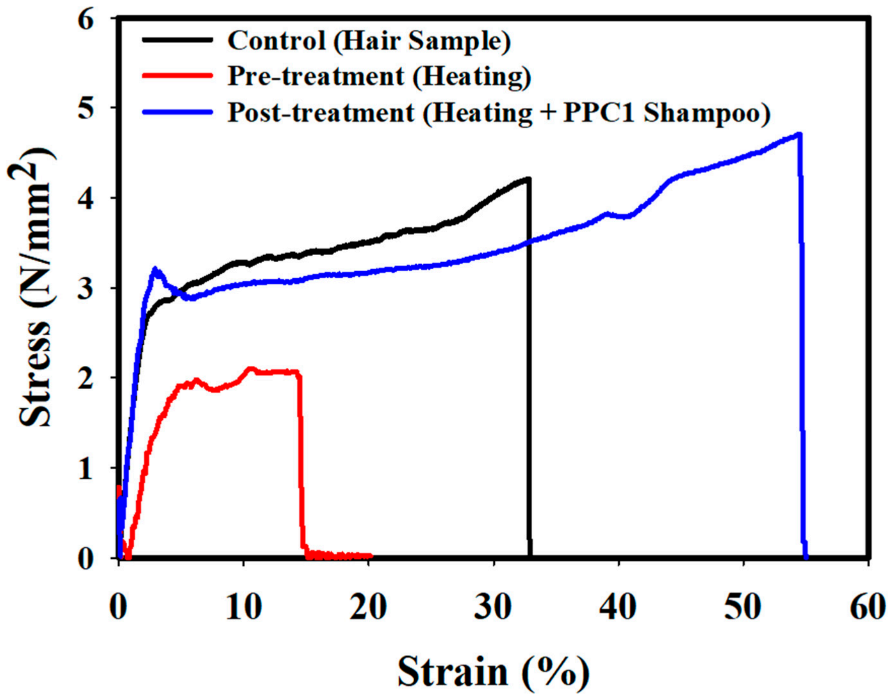

Figure S4. Tensile strength measurement of heat-damaged hair sample pre- and post- treatment with PPC1-supplemented shampoo.

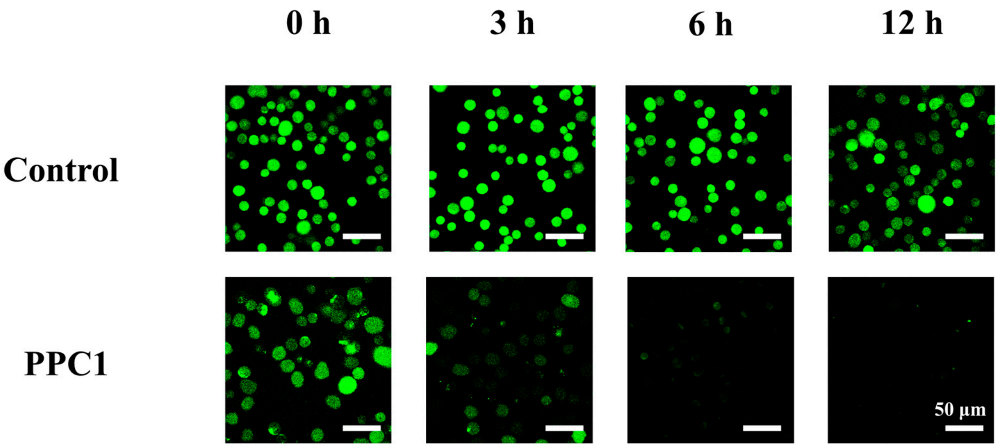

Figure S5. Confocal images of ROS staining assay using H2DCFDA stain on HeLa cells in the absence (control) and presence of PPC1.
